# Effect of increasing levels of rice distillers’ by-product on growth performance, nutrient digestibility, blood profile and colonic microbiota of weaned piglets

**DOI:** 10.5713/ajas.19.0278

**Published:** 2019-08-03

**Authors:** Oanh Nguyen Cong, Bernard Taminiau, Dang Pham Kim, Georges Daube, Giap Nguyen Van, Jérôme Bindelle, Papa Abdulaye Fall, Ton Vu Dinh, Jean-Luc Hornick

**Affiliations:** 1 Department of Veterinary Management of Animal Resources, FARAH Center, Faculty of Veterinary Medicine, University of Liège, 4000 Liège, Belgium; 2 Faculty of Animal Science, Vietnam National University of Agriculture, Hanoi Capital 100000, Vietnam; 3 Department of Food Sciences, FARAH Center, Faculty of Veterinary Medicine, University of Liège, 4000 Liège, Belgium; 4 Faculty of Veterinary Medicine, Department of Microbiology-Infectious Diseases, Vietnam National University of Agriculture, Hanoi 100000, Vietnam; 5 Animal Science Unit, GemABT, University of Liège, 5030 Gembloux, Belgium; 6 Genalyse Partner SA, Rue Hayeneux 62, B-4040 Herstal, Belgium

**Keywords:** Blood Profiles, Colonic Microbiota, Growth Performance, Rice Distillers’ By-product, Weaned Piglet

## Abstract

**Objective:**

This study was conducted to evaluate the effects of diets containing different wet rice distillers’ by-product (RDP) levels on growth performance, nutrient digestibility, blood profiles and gut microbiome of weaned piglets.

**Methods:**

A total of 48 weaned castrated male crossbred pigs, initial body weight 7.54±0.97 kg, and age about 4 wks, were used in this experiment. The piglets were randomly allocated into three iso-nitrogenous diet groups that were fed either a control diet, a diet with 15% RDP, or a diet with 30% RDP for a total of 35 days. Chromium oxide was used for apparent digestibility measurements. On d 14 and d 35, half of the piglets were randomly selected for hemato-biochemical and gut microbiota evaluations.

**Results:**

Increasing inclusion levels of RDP tended to linearly increase (p≤0.07) average daily gain on d 14 and d 35, and decreased (p = 0.08) feed conversion ratio on d 35. Empty stomach weight increased (p = 0.03) on d 35 while digestibility of diet components decreased. Serum globulin concentration decreased on d 14 (p = 0.003) and red blood cell count tended to decrease (p = 0.06) on d 35, parallel to increase RDP levels. Gene amplicon profiling of 16S rRNA revealed that the colonic microbiota composition of weaned pigs changed by inclusion of RDP over the period. On d 14, decreased proportions of *Lachnospiraceae_ge*, *Ruminococcaceae_ge*, *Ruminococcaceae_UCG*-*005*, and *Bacteroidales_ge*, and increased proportions of *Prevotellaceae_ge*, *Prevotella_2*, and *Prevotella_9* were found with inclusion of RDP, whereas opposite effect was found on d 35. Additionally, the proportion of *Lachnospiraceae_ge*, *Ruminococcaceae_ge*, *Ruminococcaceae_UCG*-*005*, and *Bacteroidales_ge* in RDP diets decreased over periods in control diet but increased largely in diet with 30% RDP.

**Conclusion:**

These results indicate that RDP in a favorable way modulate gastrointestinal microbiota composition and improve piglet performance despite a negative impact on digestibility of lipids and gross energy.

## INTRODUCTION

Rice distillers’ by-product (RDP) is a widespread coproduct from alcohol industry in Vietnam and in Asia, and is an excellent source of protein, fiber, and minerals [1–3]. It also contains other nutrients generated from fermented rice [4,5]. Owing to its cheap price it could thus help to replace more expensive feed sources for swine [3,6]. Moreover, in pig production, dietary modulation was considered as strategic ways for reducing enteric diseases, improve gut health, and enhance growth performance [7–11]. Interestingly, diets containing agro-industrial by-products from ethanol production are known to reduce diarrhea incidence in weaned piglets [12]. Furthermore, piglets fed RDP diet showed increased acetic and lactic acid content in hindgut [13], and lower populations of pathogenic bacteria in digestive tract [14]. Increase in apparent ileal digestibility (AID) of crude protein (CP) [13] was found in piglets fed RDP. Additionally, a level of 4% RDP in pig diet showed to be effective as a prebiotic including positive effect on pig performance and health [15,16]. To the best of our knowledge, to date, studies on effect of RDP on piglet performance are still limited. Moreover, no data are reported on effect of RDP on blood characteristics, and gut microbiota composition using 16S rRNA gene-based metagenomic analysis. Hence, links between growth performance, plasma parameters and intestinal microbiota are fully lacking. Thus, the main objectives of this study were to compare growth performance, nutrient digestibility, blood profiles, and colonic bacterial microbiota between weaned piglets fed diets containing increasing amounts of RDP.

## MATERIALS AND METHODS

### Animal care

In the absence of proper regulation on the use of animals for research and animal welfare during experiments in Vietnam, the protocols were carried out according to the best practices usually accepted by the Ethical Committee of University of Liège (Belgium) when conducting similar experiments.

### Rice distillers’ by-product used

The RDP originated from a glutinous rice alcohol manufacturer in the Phu Loc village, Hai Duong province in the Red River Delta region in Vietnam. The RDP was obtained from rice distillation under traditional alcohol production process. Briefly, rice was heated over steam and then spread out on a flat surface to allow cooling. Yeast was then sprinkled over rice and both were well mixed. Preparation was placed in lidded buckets for incubation for about three days. The buckets were then filled with water and held for about one week. Finally, alcohol was distilled through a covered pot and steam ducted away to a water-cooled condenser. The residues were considered as RDP for pig feeding. The RDP samples were collected immediately after alcohol distillation for chemical analysis prior to diet formulation for the trial.

### Experimental design

The study was carried out from January to March 2017 at a private farm specialized in pig production, in Hai Duong province, located 40 km from the Vietnam National University of Agriculture (VNUA), Hanoi, Vietnam. A total of 48 healthy weaned castrated male crossbred pigs (♂Duroc×♀[Landrace× Yorkshire]), originating from 12 sows to 4 male piglets per sow, 3 to 5 litter order, initial body weight (IBW) 7.54±0.97 kg (mean±standard deviation), and age about 4 wks, were used in this experiment. The piglets were individually ear tag numbered and randomly allocated into three diet-groups of four blocks according to similar IBW and sow origin by treatment. There were four replicate pens (block) per treatment and 4 pigs per concrete pen (3.6×1.1 m) with a height of 0.6 m, each pen equipped with stainless steel feeding trough and 2 automatic water-drinking nipples. In each pen, a (1.2×0.6 m) plywood piece and a 175W red infrared heat lamp bulb were used in order to maintain effective temperature for pig development. The room temperature was kept at approximately 30°C the first week, and afterwards was decreased by 1°C each week of the experiment, suitable for animal development. Piglets were fed one of three diets (a control diet without RDP, RDP0; and two experimental diets with 15%, RDP15; and 30%, RDP30; RDP on a DM basis) during a total of 35-d experiment for 2 stages (early phase, from d 0 to d 14; and late phase from d 15 to d 35). Chromium oxide (Cr_2_O_3_) added at 5 g/kg dry matter (DM) of diet was used as digesta flow marker to measure digestibility. On d 14 and d 35, two pigs per pen were randomly selected and transported 4 hours after feeding from the farm to Faculty of Veterinary Medicine, VNUA (about one hour quite travel in suitable vehicle). These pigs rested for one hour and were then killed for collection of digesta and intestinal tissue samples.

### Ingredients, diets, feeding and animal management

Raw feed ingredients were purchased all at once from a local feed company, except RDP which was delivered daily from the manufacturer. Feed was ground into flour through a 2 mm screen before mixing. The ingredients were formulated in order to obtain three iso-nitrogenous experimental diets. The RDP0 mainly consisted of corn, soybean, rice, fishmeal, and their by-products. Other diets (RDP15 and RDP30) were formulated separately with the same ingredients as the control diet, until RDP was extemporaneously included at 15% or 30% in the diets, on a DM basis (Table 1).

At each feeding, RDP0 diet was weighed and moistened with drinking water (pH 6.73) at a 1:1 ratio in order to prevent losses and to facilitate intake, and the two other ones were mixed with RDP. Prior to diet allocation, feed pH was measured, as possible covariate factor of other parameters such as palatability. Samples of RDP and complete diets at feeding were collected one time per week and stored at −20°C until the end of the experiment where they were thawed and homogenized for chemical analysis (Table 1). Daily feed allowance was divided into four equal amounts and was offered at 06:00, 11:00, 16:00, and 20:00 h during the entire experimental period. Diet amounts were increased gradually parallel to piglet development, at about 4% of live weight. Refusals were collected just before each feeding, weighed, stored at −20°C until the end of the experimental period where they were thawed and homogenized for DM analysis. Pigs always had free access to water by nipple drinkers.

### Measurements

#### Animal performance, visceral organ weight, and gastrointestinal pH

Piglets were individually weighed on d 0, d 14, and d 35 of the experiment. Average daily DM feed intake, average daily gain (ADG), and feed conversion ratio (FCR) were calculated for each pen, diet group, and each experimental period. On d 14 and d 35, the heart, liver, kidney, spleen, lung, stomach, small intestine, and large intestine of the killed pigs were immediately collected, blotted using absorbent paper, and then weighed (VIBRA balance, Tokyo, Japan). Digesta from stomach, small intestine and large intestine were carefully removed before organs weighing. The pH of stomach, ileum, caecum, and colon digesta were measured immediately after removal of segments using an electrode of a portable pH meter (HANNA, Singapore).

#### Digestibility

For apparent total tract digestibility (ATTD), fecal samples from each pen were collected two times per day for the last 5 days of the experiment and stored −80°C. At the end of the collection, the fecal samples from animals in each diet group were thawed, mixed and pooled, after which they were dried and analyzed for ATTD estimation. For AID, individual ileal digesta samples on d 35 were collected immediately post-mortem, at distal ileum about 10 cm anterior to ileo-caecal valve, and stored at −80°C until further analysis. The ileal digesta samples from animals in each diet group were then thawed, mixed and pooled, after which they were dried and analyzed for AID estimation. The AID and ATTD of nutrients were calculated relative to the chromium content using the following equation [17]:

$$\text{Nutrient digestibility }(\%\;\text{of intake})=\left[1-\frac{Cr_{2}O_{3}(diet)\times nutrient\;(ileum/feces)}{Cr_{2}O_{3}(ileum/feces)\times nutrient\;(diet)}\right]\times 100$$

where nutrient digestibility is apparent digestibility of a nutrient or energy in the diet (%); nutrient (diet) and nutrient (ileum/feces) is a nutrient (g) or energy (kcal/kg DM) concentration in the diet and the ileal/feces samples, respectively; Cr_2_O_3_ (diet) and Cr_2_O_3_ (ileum) are the Cr_2_O_3_ concentration (g/kg) in the diet and the ileal or feces samples, respectively.

#### Blood characteristics

Blood samples from two pigs per pen were collected via neck-internal jugular vein using sterile needle before morning feeding on d 14 and d 35. At each collection time, 4 mL of blood from each pig were collected into both K_2_ethylenediaminetetraacetic acid (EDTA) and serum tubes (Zhejiang Gongdong Medical Technology Co. Ltd, Zhejiang, China). The blood samples were kept in suitable ice-box and transported from the farm to analytical laboratory for maximum 60 minutes delay. The blood collection in EDTA tubes were automatically analyzed using hematology analyzer ABX Pentra DX 120c in order to determine red blood cell (RBC) and white blood cell (WBC) counts, hemoglobin (Hb), lymphocytes percentage. The blood collection serum tubes were centrifuged for 15 minutes in a bench centrifuge at 3,000 rpm, and then clean serum was collected and stored at 4°C until further analysis. The serum samples were analyzed using Cobas 8000 modular analyzer series (Roche-Hitachi, Tokyo, Japan) in order to determine biochemistry and immunology parameters including albumin, globulin, immunoglobulin G (IgG), and IgM.

#### Gut microbial composition

- Colonic digesta samples: colonic samples of 48 piglets were individually harvested after slaughter, in which 8 animals per diet on d 14 and d 35. Individual two samples of each colonic digesta were separately collected in sterile tubes (PSP Spin Stool DNA Plus Kit, Berlin, Germany), and stored at −80°C until further DNA extraction.
- DNA extraction and purification: Genomic DNA was extracted and purified from colonic digesta samples using PSP Spin Stool DNA Plus Kit (STRATEC Molecular GmnH, Berlin, Germany) following the manufacturer’s recommendations. The integrity of DNA was tested by 1% agarose gel electrophoresis. DNA concentrations were measured by absorbance at 260 nm and its purity was estimated by determining the A260/A280 ratio with using Eppendorf BioSpectrometer basic (Hamburg, Germany). Genomic DNAs were stored at −20°C, and then transported to University of Liège (Belgium) for 16S ribosomal RNA (rRNA) sequencing.
- 16S rRNA gene library construction and sequencing: The 16S polymerase chain reaction (PCR) libraries were generated for samples. PCR-amplification of the V1-V3 hypervariable region of bacterial 16S rRNA were performed using following primers (with Illumina overhand adapters), forward (5′-GAGAGTTTGATYMTGGCTCAG-3′) and reverse (5′-ACCGCGGCTGCTGGCAC-3′). Each PCR product was purified with Agencourt AMPure XP beads kit (Beckman Coulter, Pasadena, CA, USA) and submitted to a second PCR round for indexing, using Nextera XT index primers 1 and 2. After purification, PCR products were quantified using Quant-IT PicoGreen (ThermoFisher Scientific, Waltham, MA, USA) and diluted to 10 ng/μL. A final quantification, by quantitative PCR (qPCR), of each sample in the library was performed using KAPA SYBR FAST qPCR Kit (KapaBiosystems, Wilmington, MA, USA) before normalization, pooling and sequencing on a MiSeq sequencer using v3 reagents (Illumina, San Diego, CA, USA). Positive control using DNA from 20 defined bacterial species and a negative control (from the PCR step) were included in sequencing run.

Sequence reads processing was used as described previ ously [18] using MOTHUR software package v1.39.5 [19], and VSEARCH algorithm [20] respectively for alignment and clustering and chimera detection. Clustering distance of 0.03 was used for operational taxonomic unit (OTU) generation; 16S reference alignment and taxonomical assignation were based upon the SILVA database (v1.28) of full-length 16S rRNA sequences [21]. From 4,553,186 raw reads (16 samples per diet, 2 periods included d 14 and d 35, 3 diets), we obtained 3,769,161 reads after cleaning (length and sequence quality) and 3,337,068 after chimeric contaminants elimination. We retained 5,000 reads per sample as a subsampling process for OTU clustering and taxonomic assignment. Good’s coverage estimator was used as a measure of sampling effort for each sample, with a mean value of 99.70%.

Subsample datasets were used to assess alpha diversity us ing Reciprocal Simpson biodiversity index (diversity), Chao1 richness index (richness), and Simpson evenness index (evenness) at the genus level using MOTHUR. Beta diversity was assessed with MOTHUR using distance matrices based on Bray-Curtis dissimilarity index (a measure of community structure that considers shared OTUs and their relative abundances) and non-metric dimensional scaling, based upon the Bray-Curtis dissimilarity matrix was applied to visualize the biodiversity between the groups. Analysis of molecular variance test with 100,000 permutations was performed to assess the diversity clustering of treatment diets with Bray - Curtis matrix using MOTHUR [22]. Ordination analysis and 3 d plots were performed with Vegan [23], Vegan3d [24] and rgl [25] packages in R [26].

All biosample raw reads of colonic digesta samples have been deposited at the National Center for Biotechnology Information (NCBI) and are available under the Bioproject ID PRJNA428433.

### Chemical analysis

The DM of RDP, diets, and digesta isolated from ileum were dried by oven drying at 70°C for 15 h, 90°C for 5 h and 102°C for 5 h consecutively, and they were milled separately through a 1 mm screen prior to analysis. The DM of feed ingredient was determined according to Method 934.01 from AOAC [27]. Feed ingredients and diets were analyzed for CP (Method 954.01, AOAC, 1990), ether extract (EE; Method 920.39, AOAC, 1990) with petroleum ether solvent, ash (Method 942.05, AOAC, 1990), crude fiber (CF; Method 962.09, AOAC, 1990), neutral detergent fiber and acid detergent fiber (ADF) (Method 973.18, AOAC, 1990) with fiber filter bags of Ankom technology F57, phosphorus (P; Method 965.17, AOAC, 1990) using a UV-vis spectrophotometer. Calcium (Ca) was determined by titration with a standardized solution of EDTA as previously described [28], and starch was determined from estimation of reducing sugars by dinitrosalicylic acid method under spectrophotometer UV-1800 (Kyoto, Japan) as previously described [29]. Chromium was analyzed using UV absorption spectrophotometry (UV-1800, Japan) according to [30]. The GE of samples were measured using a bomb calorimeter E2K (Germany). The pH value of RDP was determined using an electrode of a portable pH meter (HANNA, Singapore). Organic acids (OA) were determined by Vinger method (Ngoan, 2002). Ethanol in RDP was determined using high performance liquid chromatography (Agilen 1200 series; Agilent Technologies, Santa Clara, CA, USA) with Aminex HPX-87H column, RI detector, phase mobile including H_2_SO_4_ 10 mM; mobile phase flow rate was 0.5 mL/min, the column temperature was maintained at 60°C.

### Statistical analysis

Data for growth performance and blood profiles were analyzed using the PROC MIXED procedure of SAS software (Version 9.4, Institute Inc., Cary, NC, USA). The statistical model included the diets (n = 3) as fixed effect, and the blocks (n = 4) as random effects. Pen was used as experimental unit for the performance data, and individual pig as experimental unit for visceral organ weights, gut pH and blood profiles. For repeated measures performed on the same experimental unit a similar model was used but including the effect of a compound symmetry structure of covariance. Orthogonal polynomials were performed to determine linear and quadratic effects of increasing level of the RDP in diets [31]. Data for pigs fed diets containing RDP were compared with data for pigs fed control diet using orthogonal contrasts. The multiple comparisons of least square means were performed according to PDIFF option. Significance was defined as p< 0.05 and 0.05<p<0.10 was considered as a trend. Due to unintentional feces and digesta pooling by diet, no statistical analysis could be performed on digestibility data.

Data for microbiota composition in the colon, the statis tical differences in bacterial diversity, bacterial richness and bacterial evenness between pig fed control diet and diets containing RDP were performed using Kruskal-Wallis with Benjamini, Krieger, and Yekutieli test (PRISM 7.0, Graph-Pad software, La Jolla, CA, USA). Beta diversity (Bray-Curtis dissimilarity) was compared using Kruskal-Wallis with Bonferroni correction (p≤0.003). To compare statistical differences in bacterial community abundance between diets, a non-parametric Kruskal-Wallis H tests was combined to a Storey False Discovery Rate followed by Tukey-Kramer post-hoc test (using STAMP 2.1.3 software) as previously described [32]. The significant level used for statistical tests was 0.05.

## RESULTS

### Animal performance, visceral organ weight, pH value of digestive tract and digestibility

With inclusion levels of RDP, a linear trend (p = 0.07) for increasing ADG was observed in the two experimental periods, and FCR tended to decrease in the second one (p = 0.08) (Table 2). Final body weight (FBW) at d 35 thus increased linearly with dietary RDP inclusion. Besides, using orthogonal contrasts, increase in FBW was observed (p<0.05) when pigs fed RDP diets were compared with control pigs. As a rule, no significant differences in visceral organ weights were observed between groups, but heart weight at d 14 and empty stomach weight at d 35 were negatively and positively impacted, respectively, by RDP level (p = 0.04 and p = 0.03) (Table 3). With level of RDP incorporation, weights of visceral organs—except gut-relative to live weight decreased on d 14 so far that their sum decreased by 4.4 and 11.6%, in RDP15 and RDP30, respectively, when compared to the control group.

When regard to pH values of stomach, ileum, caecum and colon contents, no differences between groups were observed at both d 14 and d 35. However, the pH values decreased numerically in all segments at d 14 when increasing inclusion of RDP in diet (Table 4).

**Figure 1:**
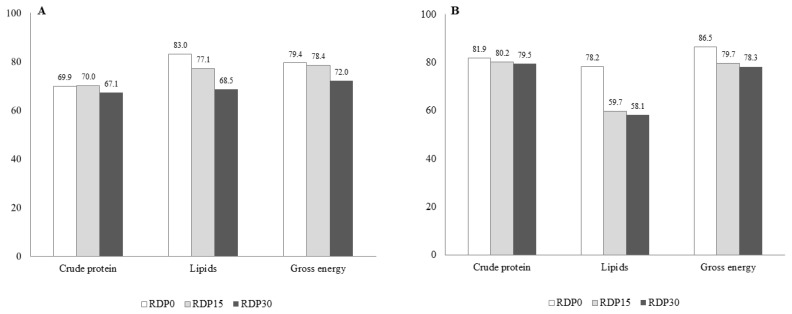
Coefficients of apparent ileal digestibility (AID) (A) and of apparent total tract digestibility (ATTD) (B) of weaned pigs fed diets containing different levels of rice distillers’ by-product. RDP0, control diet; RDP15, diet with rice distillers’ by-product accounting for 15% dry mater; RDP30: diet with rice distillers’ by-product accounting for 30% dry mater.

The AID and ATTD of CP, lipids, and gross energy numer ically decrease with inclusion of RDP, especially for energy and lipids (Figure 1).

### Physical and chemical properties of the blood

On d 14, a linear decrease in amount of globulin was observed (p = 0.003) in pigs fed RDP diets when compared with pigs fed control diet. Quadratic effects were observed (p≤0.04) for WBC, lymphocyte, and albumin. On d 35, a trend for linear effect for RBC (p = 0.06) and a trend for quadratic effect for Hb (p = 0.09) were observed between diet groups. Other hematological parameters were not affect by pigs fed RDP diets compared with pigs fed control diet (Table 5).

### Colonic microbiome analysis by 16S rRNA profiling

**Figure 2:**
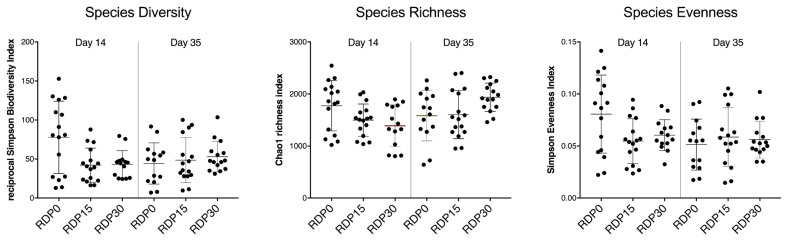
Alpha diversity comparisons for colon composition of weaned piglets fed rice distillers’ by-product levels on d 14 and d 35 detected by 16S rRNA gene metagenetic analysis.

The analysis of alpha diversity of colonic bacterial population showed that no statistical differences were found (p>0.05) between weaned pigs fed RDP diets and pigs fed control diet within the same period, as estimated by diversity indices including reciprocal Simpson Biodiversity, Chao1 richness, and Simpson Evenness (Figure 2). However, bacterial richness of colonic microbiota was greater in RDP30 on d 35 than on d 14 (p = 0.01). Beta diversity differed between diet groups as indicated by clear clustering on principal coordinates of microbial profiles on d 14 and d 35. Indeed, colonic microbiota communities differed between RDP0 and RDP30 on d 14 and d 35 (p = 0.002, and p<0.001), and between RDP15 and RDP30 on d 35 (p = 0.002).

**Figure 3:**
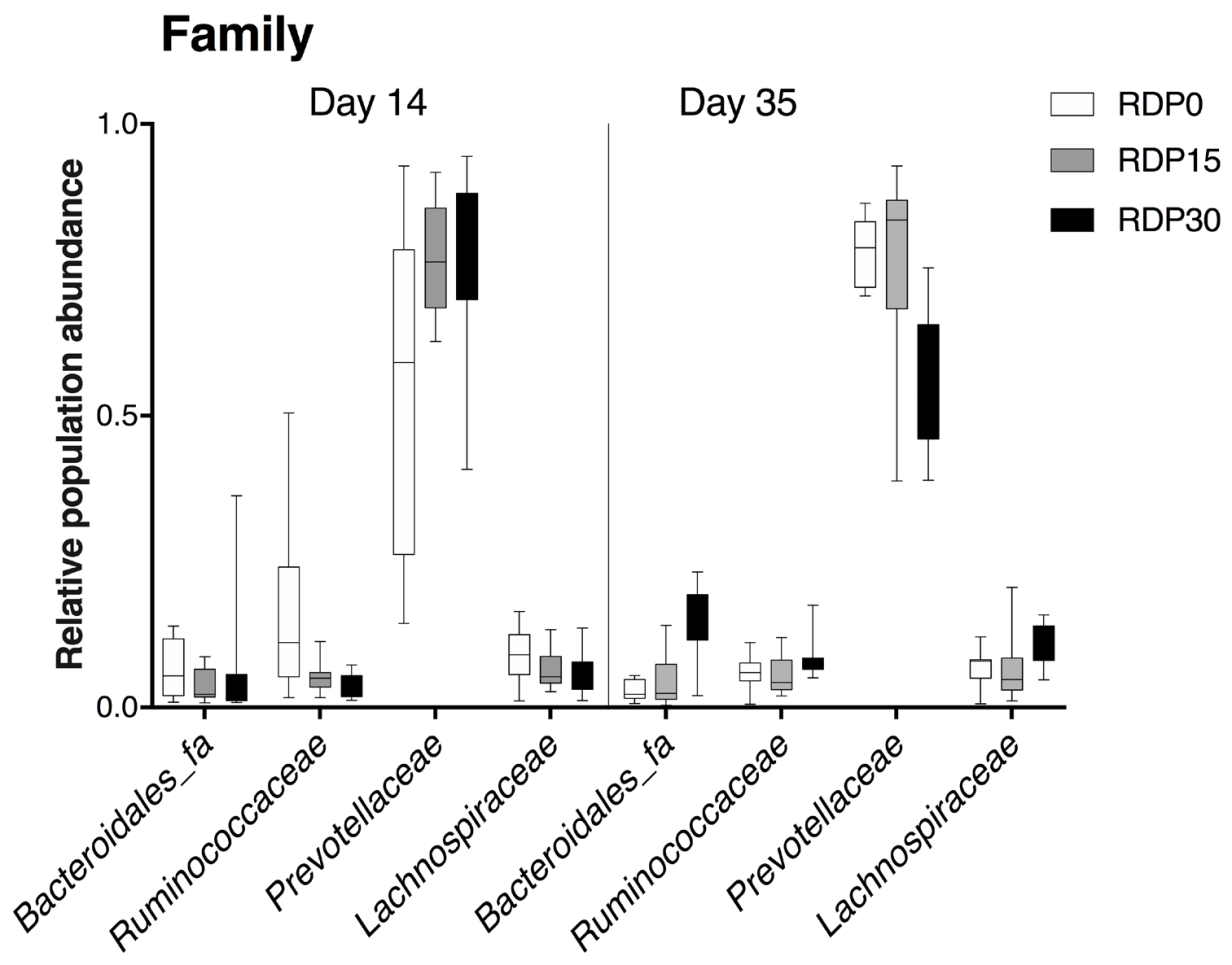
Differences in relative abundance at the family level of colonic microbiota of weaned pigs fed diets containing different levels of rice distiller’ by-product on d 14 and d 35 detected by 16S rRNA gene metagenetic analysis. RDP0, control diet; RDP15, diet with rice distillers’ by-product at 15% dry matter; RDP30, diet with rice distillers’ by-product at 30% dry matter.

**Figure 4:**
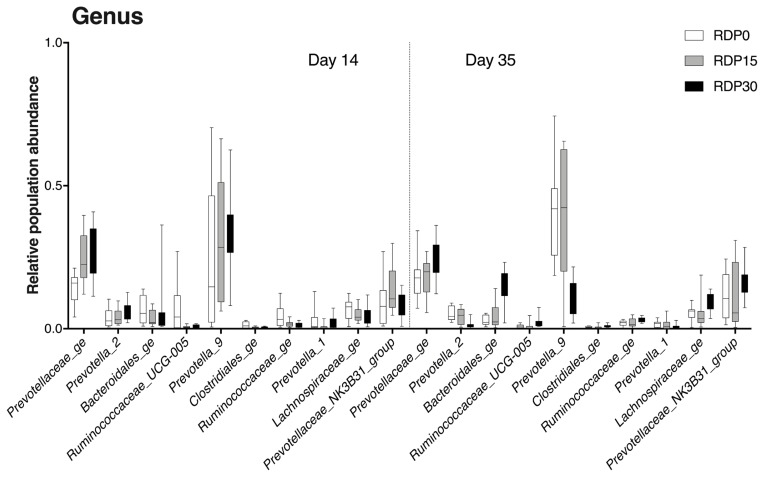
Differences in relative abundances at the genus level of colonic microbiota of weaned pigs fed diets containing different levels of rice distiller’ by-product on d 14 and d 35 detected by 16S rRNA gene metagenetic analysis. RDP0, control diet; RDP15, diet with rice distillers’ by-product at 15% dry matter; RDP30: diet with rice distillers’ by-product at 30% dry matter.

Differences in colonic bacterial compositions at taxonomic levels (family and genus) are presented in Figure 3 and 4. When comparing relative abundance of family of diets in a contemporary, proportion of Lachnospiraceae in RDP30 was lower on d 14 (p = 0.002) and greater on d 35 (p<0.001). Proportion of Ruminococcaceae was greater in RDP0 (p<0.001) on d 14, whilst greater in RDP30 on d 35 (p≤0.03). Proportion of Bacteroidales_fa was lower in RDP15 on d 14 (p≤0.04), and greater in RDP30 (p<0.001) on d 35. And proportion of Prevotellaceae increased (p≤0.01) on d 14 and decreased (p≤0.05) on d 35 according to inclusion levels of RDP (Supplementary Table S1).

When comparing relative abundance of genus of diets in a contemporary, *Lachnospiraceae_ge* in RDP30 was respectively lower (p = 0.008) and greater (p<0.001) on d 14 and d 35. *Ruminococcaceae* spp. was greater in RDP0 on d 14, while greater in RDP30 on d 35 (p<0.001). *Bacteroidales* was lower in RDP15 on d 14 (p≤0.002), whilst greater in RDP30 on d 35 (p<0.001). *Prevotellaceae* increased with inclusion of RDP on both d 14 and d 35 (p<0.001). *Prevotellaceae_NK3B31_group* was greater in RDP15 on d 14 (p≤0.001) and in RDP30 on d 35 (p<0.001). *Prevotella_1* was lower in DAR15 on d 14 (p≤0.02). Increases in *Prevotella_2* and *Prevotella_9* on d 14 (p≤0.04) and an inverse tendency on d 35 (p<0.001) were found with inclusion level of RDP (Supplementary Table S2).

## DISCUSSION

### Chemical composition of the diets

Rice by-products classically are incorporated in diets for pig in Asia. Thus, one could argue some confounding effects between RDP levels and the other rice ingredients of the diets. Confounding bias cannot be completely avoided in such experiments. The main constraint aimed to preserve iso-nitrogenous characteristics of the diets. The RDP showed a high CP level, this constraint only could be fulfilled thanks to decreases in soybean by-products levels. With CF content close to 0.35%, broken rice could be considered to maintain similar levels of starch between the groups. Rice bran helped to compensate for CF provided by RDP. In this context, it should be kept in mind that the level of RDP incorporation in the diets was the main—and thus not the exclusive—factor of variation.

### Animal performance, visceral organ weight, and pH value of digestive tract

In the first three days of the experiment, loose feces appeared on some experimental pens due to digestive disorders, this phenomenon resolved few days after feed intake amount was reduced. Therefore, diarrhea in piglets was not evaluated during the entire experimental period. All piglets in our experiment remained in good health, probably due to a good set-up with clean environment during the experimental period. Increasing inclusion level of RDP did not affect DM intake, and tended to improve ADG during d 0 to d 14 and d 15 to d 35, leading to higher final live weights in animals fed RDP. Interestingly, feed efficiency was improved only from d 15 to d 35. According to earlier studies, piglets fed diets with 10%, 15%, 20%, and 30% RDP had enhanced ADG and FCR compared with piglets fed control diet, but without effect in DM intake during a 42-d experiment [2,13], suggesting that RDP does not affect diet palatability in weaned pigs. Lower pH and higher lactic and acetic contents in RDP product could be beneficial to the gut health of piglets through a decrease in number of harmful bacteria such as *Escherichia coli* and total coliforms, and an increase in number of beneficial bacteria such as lactic acid bacteria [13]. This has been shown for pigs fed fermented diets [13,33]. In addition, short chain OA, mainly acetate, propionate, butyrate and lactate are produced in large intestine by fermentation of undigested protein and fiber fractions. These OA had deep effects on metabolism and gut health [34]. Acetate and propionate are energy substrates for lipogenesis and gluconeogenesis, respectively, and butyrate is used primarily by colonocytes as major energy source for their metabolic activities in pigs [35–38]. Hong et al [13] demonstrated that the concentrations of acetic acid and lactic acid in colonic digesta were greater for piglets fed RDP diet than those fed diet without RDP. Thus, our results suggest that the positive effect on growth performance of weaned piglets fed RDP diets may be associated with modulation of OA-production bacteria due to various diet components. In addition, no differences in almost all visceral organ weights among diet groups was observed. This is similar to previous report from [6] who demonstrated that there were no differences in heart, lung, liver, spleen, stomach, kidney, small intestine, and large intestine weights in growing pigs fed diets with different inclusion levels of RDP (0%, 7.5%, 15%, 22.5%, 30% DM). However, an increase in relative weight of empty stomach on d 35 was found with RDP diets, which could be due to soluble fiber content of RDP originated from glutinous rice [39] leading to increase in transit time of diets [40] from stomach to intestine. This could increase digestive fluids secretion and activity for breaking down feed that resulted in stomach enlargement [41]. Lower heart weight of pigs fed RDP diets on only d 14 is unclear and possibly due to fortuitous statistical significance but the more than 4% to 11% numerical decreases of the overall visceral—but stomach and intestines—proportions of live weight is noticeable. Such decreases suggest a more efficient viscera metabolism, may be consecutive to ready-to-use metabolites stemming from fiber fermentation, reducing thus metabolic burden for these organs. This hypothesis would merit to be further investigated.

### Digestibility

Recent study [13] reported that a greater AID and a similar ATTD of CP was found for weaned pigs fed diet with 20% RDP than pigs fed control diet for a 42-d experiment. Moreover, piglets fed pectin-containing diet showed higher digesta viscosity with decrease ileal digestibility of protein [42]. Thus, and in the limit of variance indicators failure, the numerical decrease in AID and ATTD of CP in RDP diets could be related to high pectin content. The decrease in ATTD of CP with RDP inclusion could indicate a partial nitrogen flux shift from plasma (urea) to hindgut lumen, thanks to nitrogen conversion to microbial protein, the one thereafter excreted in feces. Additionally, a decrease in AID and ATTD of EE with inclusion of RDP, as observed in this study, has never been reported. According to [43] piglet diets supplemented with pectin could have negative effect on fat digestibility. A other study [44] revealed that weaning pigs fed diets containing OA had changed intestinal microbiota composition, with decreased amount of bile acid production, thus leading to negative impact on fat digestion. The OA issued from RDP diets possibly contributed to alter EE CATTD.

### Physical and chemical properties of the blood

Effect of RDP incorporation in weaned pig diet on blood profiles has not been published yet. This study found no effect of RDP on hematological profiles such as WBC, Hb, and lymphocyte percentage, and biochemical parameters such as serum concentrations of albumin, IgG, and IgM, except serum globulin concentration on d 14 and RBC count on d 35. Despite decreased RBC and globulin values with inclusion of RDP, all results were found to be within the normal ranges for weaned pigs, as reported previously [45–48]. The difference in values over time were considered as normal owing to the pig developmental stage [40]. Additionally, according to [44], IgA, IgG, and IgM concentrations were not influenced by supplementation of OA in weaning piglet diets, which in line with our results. The largest immunologically competent organ in the body is gut or its associated lymphoid system, and the development of immune system is associated with composition of indigenous micro flora [49]. With inclusion of RDP, the decrease in serum globulin concentration on d 14 and of RBC count on d 34 was not clear and could be due to stochastic or incidental effects without relationships with RDP. Further studies are required to drain more conclusion.

### Colonic microbiome analysis by 16S rRNA profiling

Intestinal microbiota, that affect nutrient metabolism and immune system development, play a significant role in intestine and host health of pigs [50]. Gut microbiota composition is affected by diet composition, in particular fiber components [51]. In this study, we compared the microbial population structure and microbiota composition from colonic digesta of the weaned pigs. To the best of our knowledge, this is the first report to evaluate the effect of RDP inclusion on the colonic microbiota of weaned pigs using 16S rRNA gene sequence analysis. On d 35, colonic microbial richness increased in piglets fed RDP30 in the later phase of feeding trial, suggesting that inclusion of 30% RDP may stimulate the growth of colonic bacteria in a long-term intervention. Moreover, beta diversity analysis indicated different clustering of microbial community structure between RDP diets and control diet. In addition, the microbial compositions significantly varied with inclusion of RDP at family and genus levels. The change in relative abundance of colonic microbiota composition could be related to the chemical components of RDP, especially fiber fraction. Most of the time, the composition of bacterial communities in colonic contents presented opposite evolution when comparing d 14 and d 35 values. This could due to an adaption phase of immature digestive tract of weaned piglets. An increase in relative abundance of *Prevotellaceae_NK3B31_group* was found in pigs fed diets containing high component of arabinoxylan on d 28 [52], which also was found in our results. Besides that, a considerable decrease in *Prevotella_1*, *Prevotella_2*, and *Prevotella_9* proportions was observed in piglets fed RDP30 on d 35. The reason for this variation might be associated with increased microbiota compositions of *Prevotellaceae_ge*, *Prevotellaceae_NK3B31_group*. Indeed, arabinoxylan fiber, an important content of plan cell walls of rice, is considered as a prebiotic effect on intestinal health of pigs stimulating the growth of the latter bacteria genera [53,54]. Moreover, members of Lachnospiraceae family —whose proportion increased at d 35 in our experiment—was positively correlated with intestinal epithelial cell energy metabolism and butyrate production [55, 56]. *Ruminococcacecae* spp. also was related to butyrate production [55]. Finally, a higher abundance of *Bacteroides* spp., *Lachnospiraceae* spp., and *Ruminococcacecae* spp. was linked to obese animals [57–59]. All these arguments are in line with the better performances observed at the end of the experimental period in our experiment. Also, piglets fed RDP diet had a higher acetic acid concentration in colon [13], and butyrate-producing bacteria are able to use acetate [60]. For example, a positive correlation between concentration of acetic acid and butyric acid in colon of pigs fed diets with increasing inclusion of chicory forage was reported by a previous finding [60], and [61] reported that OA induced adipose phenotypes. Thus, RDP diets alter not only composition of colonic microbiota but also microbiota metabolism. In addition, [51] found that microbiota can improve host fat storage. The OA controlled metabolic regulation by signal via G-protein-coupled receptors (GPCRs), such as GPR41-relative to the adipose tissues, and effects of the microbiota on fat deposition depends on this OA receptor [62]. However, further studies are needed to clarify the effects of microbiota composition on colonic gene expression profiling.

## CONCLUSION

This study indicated that weaned pigs fed RDP modulated the gastrointestinal microbiota composition over the period, especially at the highest inclusion levels, with higher abundance of population bacteria susceptible to improve fat deposition and animal performance. Negative effects of RDP on digestibility of several nutrients should be confirmed but, in the case of nitrogen, could be reflect shift in nitrogen flux. The effects of RDP on some plasma parameters have to be taken with caution due to their normal value ranges. Further studies are needed to highlight possible relationships between RDP-associated gut microbiota metabolism and pig growth physiology.

## Figures and Tables

**Table 1:** Ingredients and nutrient composition of the experimental diets

Items	Dietary treatment1)		

	RDP0	RDP15	RDP30
Ingredients (% DM)			
Corn 7.5% CP	9	13	15
Corn thermally treated	24	30	31
Soybean meal 46% CP	10	5	2
Soybean full fat extruded 36% CP	20	15	7.85
Fishmeal 60% CP	5	5	5
Broken rice	20.9	9.85	5
Rice bran (full fat)	9	5	2
Rice distillers’ by-product2)	0	15	30
Limestone 38%	1.2	1.2	1.2
Cr_2_O_3_	0.5	0.5	0.5
Salt	0.2	0.2	0.2
Vitamin mineral premix3)	0.25	0.25	0.25
Analyzed composition (% DM) and energy value (MJ/kg DM)			
Dry matter	38.6	38.2	24.0
Crude protein	24.1	24.0	23.9
Ether extract	5.87	6.26	8.09
Ash	6.43	5.61	5.38
Crude fiber	4.17	4.20	5.26
Neutral detergent fiber	40.7	43.1	37.8
Acid detergent fiber	6.64	7.87	6.43
Starch	48.5	45.0	44.5
Calcium	0.83	0.73	0.65
Total phosphorus	0.61	0.65	0.70
Gross energy (MJ/kg DM)	19.1	19.4	19.7
Metabolisable energy4) (MJ/kg DM)	15.5	16.0	16.1
Lysine	1.29	1.17	1.07
Methionine	0.42	0.47	0.53

DM, dry matter; CP, crude protein.

1) RDP0, control diet; RDP15, diet with rice distillers’ by-product accounting for 15% DM; RDP30, diet with rice distillers’ by-product accounting for 30% DM.

2) Analyzed data (% DM): dry matter, 8.12; crude protein, 35.28; ether extract, 0.7; neutral detergent fiber, 28.10; acid detergent fiber, 16.50; calcium, 0.14; total phosphorus, 0,42; gross energy (MJ/kg DM), 12.83; pH, 3.07; lactic, acetic, and butyric acid (g/100 g fresh sample), 2.07, 0.03, 0.04; ethanol (mg/kg fresh sample), 6.2.

3) Premix contains (each kg of premix): vitamin A, 6,000,000 IU; vitamin D_3_, 800,000 IU; vitamin E+polyphenols, 20,000 mg; vitamin E, 15,000 mg; niacin, 10,000 mg; acid pantothenic, 4,000 mg; vitamin B_2_, 1,600 mg; vitamin K_3_, 800 mg; vitamin B_1_, 400 mg; vitamin B_6_, 400 mg; axit foric, 400 mg; biotin, 40,000 mcg; vitamin B_12_, 8,000 mcg; Zn, 100,000 to 110,000 mg; Cu, 64,000 to 70,400 mg; Fe, 48,000 to 52,800 mg; Mn, 24,000 to 26,400 mg; I, 1,600 to 1,760 mg; Se, 120 to 132 mg; moisture, 10%.

4) Calculated data according to equation of [63] for ME estimation: ME = 4,168–12.3× Ash+1.4×CP+4.1×EE–6.1×CF (g/kg DM).

**Table 2:** Dry mater intake, average daily gain, and feed conversion ratio (LSM) of piglets fed diets containing different levels of rice distillers’ by-product

Items	Treatment1)			SEM	p-value	
	
	RDP0	RDP15	RDP30		Linear	Quadratic
d 0 to d 14						
Number of animals	16	16	16			
IBW (kg)	7.55	7.53	7.53	0.51	0.87	0.96
FBW (kg)	10.2	10.4	10.5	0.61	0.23	0.84
DMI (g/d)	402	401	399	30.6	0.18	0.59
ADG (g/d)	190	204	212	9.44	0.07	0.74
FCR (kg/kg)	2.14	1.94	2.10	0.14	0.80	0.25
d 15 to d 35						
Number of animals	8	8	8			
IBW (kg)	10.2	10.5	10.6	0.54	0.36	0.86
FBW (kg)	19.0	20.0	20.9	0.71	0.01	0.98
DMI (g/d)	723	722	724	35.4	0.42	0.35
ADG (g/d)	418	453	492	18.0	0.06	0.95
FCR (kg/kg)	1.73	1.61	1.48	0.09	0.08	1.00

LSM, least squares means; SEM, standard error of the mean; d, day; IBW, initial body weight; FBW, final body weight; ADG, average daily gain; DMI, daily dry matter intake; FCR, feed conversion ratio (kg DM feed/kg gain); DM, dry matter.

1) RDP0, control diet; RDP15, diet with rice distillers’ by-product accounting for 15% DM; RDP30, diet with rice distillers’ by-product accounting for 30% DM.

**Table 3:** Visceral organ weight (LMS) of piglets fed diets containing different levels of rice distillers’ by-product

Items	Dietary treatment1)			SEM	p-value	
	
	RDP0	RDP15	RDP30		Linear	Quadratic
Organ weight (g) on d 14						
Heart	55.6	49.9	48.2	3.58	0.04	0.50
Kidney	62.3	62.1	54.8	4.22	0.16	0.96
Liver	235	226	217	9.12	0.16	0.96
Lung	127	130	115	11.6	0.32	0.41
Spleen	18.1	17.6	18.2	1.81	0.95	0.75
Empty stomach	75.7	81.2	81.6	4.78	0.32	0.61
Empty small intestine	400	411	396	26.5	0.92	0.68
Empty large intestine	126	112	138	13.7	0.46	0.18
Organ weight (g) on d 35						
Heart	80.1	81.2	83.9	7.39	0.62	0.91
Kidney	83.8	79.9	82.5	7.02	0.84	0.57
Liver	372	377	382	24.7	0.67	0.98
Lung	176	174	172	11.4	0.79	0.99
Spleen	25.4	27.3	26.3	2.33	0.72	0.50
Empty stomach	124	133	148	7.08	0.03	0.70
Empty small intestine	671	638	652	49.4	0.65	0.53
Empty large intestine	225	216	245	25.8	0.41	0.38

LSM, least squares means; SEM, standard error of the mean; d, day; DM, dry matter.

1) RDP0, control diet; RDP15, diet with rice distillers’ by-product accounting for 15% DM; RDP30, diet with rice distillers’ by-product accounting for 30% DM.

**Table 4:** pH values (LSM) of stomach, ileum, caecum, and colon digesta of piglets fed diets containing different levels of rice distillers’ by-product

Items	Dietary treatment1)			SEM	p-value	
	
	RDP0	RDP15	RDP30		Linear	Quadratic
d 14						
Number of animals	8	8	8			
Stomach	3.68	3.27	3.21	0.32	0.17	0.55
Ileum	6.70	6.66	6.62	0.10	0.33	0.93
Caecum	5.63	5.58	5.56	0.09	0.58	0.87
Colon	5.96	5.80	5.77	0.11	0.24	0.64
d 35						
Number of animals	8	8	8			
Stomach	3.63	3.66	3.77	0.14	0.50	0.82
Ileum	6.57	6.53	6.67	0.05	0.13	0.13
Caecum	5.74	5.83	5.75	0.06	0.83	0.24
Colon	5.80	5.97	5.84	0.09	0.77	0.16

LSM, least squares means; SEM, standard error of the mean; d, day; DM, dry matter.

1) RDP0, control diet; RDP15, diet with rice distillers’ by-product accounting for 15% DM; RDP30, diet with rice distillers’ by-product accounting for 30% DM.

**Table 5:** Parameters of biochemistry, hematology, and immunology (LSM) of piglets fed diets containing different levels of rice distillers’ by-product

Items	Dietary treatment1)			SEM	p-value	
	
	RDP0	RDP15	RDP30		Linear	Quadratic
d 14						
Number of animal	8	8	8	-	-	-
WBC (Giga/L)	17.5	20.6	17.6	1.39	0.94	0.04
RBC (Tera/L)	5.59	5.64	5.94	0.25	0.34	0.70
Hb (g/dL)	10.6	10.5	11.1	0.32	0.27	0.36
Lymphocyte (%)	50.7	44.9	54.2	2.78	0.25	0.01
Albumin (ALB, g/L)	30.0	27.8	30.8	1.41	0.42	0.01
Globulin (g/L)	14.9	13.3	10.4	0.96	0.003	0.54
IgG (mg/dL)	242	233	235	14.7	0.66	0.67
IgM (mg/dL)	38.4	46.1	40.6	7.11	0.82	0.41
d 35						
Number of animal	4	4	4	-	-	-
WBC (Giga/L)	22.0	19.2	19.0	2.71	0.46	0.71
RBC (Tera/L)	6.67	6.73	6.23	0.27	0.06	0.16
Hb (g/dL)	11.9	12.1	11.5	0.31	0.18	0.09
Lymphocyte (%)	42.8	46.4	49.8	5.11	0.37	0.99
Albumin (ALB, g/L)	28.5	26.7	27.9	1.72	0.76	0.34
Globulin (g/L)	16.8	18.2	16.8	0.74	1.00	0.17
IgG (mg/dL)	85.5	88.1	85.7	3.63	0.98	0.60
IgM (mg/dL)	35.1	41.7	35.5	6.62	0.96	0.43

LSM, least squares means; SEM, standard error of the mean; WBC, white blood cell count; RBC, red blood cell count; Hb, hemoglobin; Ig, immunoglobulin; d, day; DM, dry matter.

1) RDP0, control diet; RDP15, diet with rice distillers’ by-product accounting for 15% DM; RDP30, diet with rice distillers’ by-product accounting for 30% DM.
