# Supplementary material for: Effect of increasing levels of rice distillers’ by-product on growth performance, nutrient digestibility, blood profile and colonic microbiota of weaned piglets
Source: Asian-Australas J Anim Sci. 2019 Aug 3;33(5):788–801. doi: 10.5713/ajas.19.0278 (PMC7206405; doi:10.5713/ajas.19.0278)
Supplement: Supplementary file 1 [file ajas-19-0278-suppl.pdf]

Supplementary Table S1. Differences in relative abundance at the family level of colonic microbiota (percentage) of weaned pigs fed diets containing different levels of rice distiller' by-product

Family	Day 14 (n = 16)			Day 35 (n = 16)			P-value
	RDP0	RDP15	RDP30	RDP0	RDP15	RDP30	
Lachnospiraceae	8.83^{b}	6.62^{bc}	5.70^{c}	6.67^{bc}	6.67^{bc}	10.78^{a}	0.025
Ruminococcaceae	16.5^{a}	5.08^{c}	3.80^{c}	5.89^{c}	5.53^{c}	8.35^{b}	0.002
Bacteroidales_fa	6.50^{b}	3.53^{c}	5.93^{b}	2.86^{c}	4.10^{c}	14.57^{a}	0.002
Prevotellaceae	55.2^{c}	75.9^{b}	78.6^{a}	78.2^{a}	75.9^{b}	54.8^{c}	0.002

Values are expressed as mean. Values followed by different letters in the same row indicate statistical differences ($p < 0.05$); n: number of colonic samples; RDP0: control diet; RDP15: diet with rice distillers' by-product accounting for 15% DM; RDP30: diet with rice distillers' by-product accounting for 30% DM.

Supplementary Table S2. Differences in relative abundances at the genus level of colonic microbiota (percentage) of weaned pigs fed diets containing different levels of rice distiller' by-product

Genus	Day 14 (n = 16)			Day 35 (n = 16)			P-value
	RDP0	RDP15	RDP30	RDP0	RDP15	RDP30	
Lachnospiraceae_ge	6.75^{b}	5.03^{bc}	4.58^{c}	5.39^{bc}	5.29^{bc}	8.97^{a}	0.023
Ruminococcaceae_ge	4.70^{a}	1.75^{b}	1.32^{b}	1.98^{b}	1.96^{b}	3.04^{ab}	0.008
Ruminococcaceae_UCG-005	7.06^{a}	0.63^{b}	0.76^{b}	0.91^{b}	0.83^{b}	2.24^{b}	0.003
Bacteroidales_ge	6.50^{b}	3.53^{c}	5.93^{b}	2.86^{c}	4.10^{c}	14.6^{a}	0.002
Prevotellaceae_ge	13.9^{c}	24.4^{a}	25.8^{a}	17.9^{b}	18.4^{b}	25.1^{a}	0.002
Prevotellaceae_NK3B31_group	8.89^{d}	14.0^{b}	8.41^{d}	11.4^{c}	10.6^{c}	16.2^{a}	0.068
Prevotella_1	2.94^{a}	0.69^{b}	2.64^{a}	1.73^{ab}	1.41^{ab}	0.72^{b}	0.017
Prevotella_2	3.68^{b}	4.03^{b}	5.89^{a}	5.14^{a}	4.21^{b}	1.34^{c}	0.002
Prevotella_9	24.1^{d}	30.9^{c}	33.9^{b}	41.3^{a}	39.7^{a}	10.2^{e}	0.003

Values are expressed as mean. Values followed by different letters in the same row indicate statistical differences ($p < 0.05$); n: number of colonic samples; RDP0: control diet; RDP15: diet with rice distillers' by-product at 15% DM; RDP30: diet with rice distillers' by-product at 30% DM.
